# Ungulate Reproductive Parameters Track Satellite Observations of Plant Phenology across Latitude and Climatological Regimes

**DOI:** 10.1371/journal.pone.0148780

**Published:** 2016-02-05

**Authors:** David C. Stoner, Joseph O. Sexton, Jyoteshwar Nagol, Heather H. Bernales, Thomas C. Edwards

**Affiliations:** 1 Department of Wildland Resources, Utah State University, Logan, Utah, United States of America; 2 Global Landcover Facility, Department of Geographical Sciences, University of Maryland, College Park, Maryland, United States of America; 3 Utah Division of Wildlife Resources, Salt Lake City, Utah, United States of America; 4 U.S. Geological Survey, Utah Cooperative Fish and Wildlife Research Unit, Logan, Utah, United States of America; Université de Sherbrooke, CANADA

## Abstract

The effect of climatically-driven plant phenology on mammalian reproduction is one key to predicting species-specific demographic responses to climate change. Large ungulates face their greatest energetic demands from the later stages of pregnancy through weaning, and so in seasonal environments parturition dates should match periods of high primary productivity. Interannual variation in weather influences the quality and timing of forage availability, which can influence neonatal survival. Here, we evaluated macro-scale patterns in reproductive performance of a widely distributed ungulate (mule deer, *Odocoileus hemionus*) across contrasting climatological regimes using satellite-derived indices of primary productivity and plant phenology over eight degrees of latitude (890 km) in the American Southwest. The dataset comprised > 180,000 animal observations taken from 54 populations over eight years (2004–2011). Regionally, both the start and peak of growing season (“Start” and “Peak”, respectively) are negatively and significantly correlated with latitude, an unusual pattern stemming from a change in the dominance of spring snowmelt in the north to the influence of the North American Monsoon in the south. Corresponding to the timing and variation in both the Start and Peak, mule deer reproduction was latest, lowest, and most variable at lower latitudes where plant phenology is timed to the onset of monsoonal moisture. Parturition dates closely tracked the growing season across space, lagging behind the Start and preceding the Peak by 27 and 23 days, respectively. Mean juvenile production increased, and variation decreased, with increasing latitude. Temporally, juvenile production was best predicted by primary productivity during summer, which encompassed late pregnancy, parturition, and early lactation. Our findings offer a parsimonious explanation of two key reproductive parameters in ungulate demography, timing of parturition and mean annual production, across latitude and changing climatological regimes. Practically, this demonstrates the potential for broad-scale modeling of couplings between climate, plant phenology, and animal populations using space-borne observations.

## Introduction

Vegetation phenology is an expression of climatic norms, and functions as the primary environmental cue setting the rhythm of ungulate life history events such as migration and reproduction [1–5]. In temperate regions, climate influences population growth through its effects on primary production [6], with forage quality and abundance during the perinatal period being a primary factor limiting juvenile production and survival [7, 8]. Thus, understanding the couplings between plant phenology and consumer life history characteristics may help explain systematic trends in ungulate demography [9–12].

Reproduction (defined here as the period from conception to the end of lactation) is the most energetically demanding life stage for female ungulates, and the most costly part of this cycle is the interval from late pregnancy through lactation [13]. Nutritional state of the mother during conception, lactation, and weaning impacts neonatal survival rates [8, 14]. Timing of parturition is also critical, as individuals born late in the growing season are smaller at the onset of winter, have accumulated less body fat, and exhibit relatively high surface-to-volume ratios, all of which can influence overwinter survival [15].

Given these constraints, ungulates try to match energetic demands to seasonal forage availability. They maximize the probability of conception by breeding in autumn when females have weaned offspring and are in peak physical condition [13]. Parturition and lactation, however, are timed to capture the period of maximum plant quality and productivity, which optimizes juvenile nutrition and therefore survival [2, 13, 16].

Climate and weather are the major synoptic factors affecting forage characteristics and thereby ungulate populations [6, 8]. Climatological factors also affect the timing of migration [17, 18], which can limit the length of time neonates have on summer range. Plant phenology varies annually, which can result in poor synchrony between parturition and forage availability in any given year. Under these conditions neonate nutrition can be low or variable, resulting in susceptibility to disease and predation [8]. Among conspecific ungulates geographic differences in juvenile production and survival have been noted [19–21]. Explanations implicate variation in climatological factors such as drought and winter severity; yet our understanding of the relative costs and benefits associated with timing parturition to plant phenology across contrasting climatic regimes remains poorly understood, in part because of the constraints imposed by migration in seasonal environments [22, 23].

Changes in ungulate abundance and migration behavior have been linked to climate and land-use [24, 25]. The Southwest contains some of the driest ecoregions in North America [26], and regional projections suggest further drying combined with habitat fragmentation as human population growth drives demand for water, housing, and expansion of transportation infrastructure [27, 28]. Therefore, understanding the phenological underpinnings of ungulate reproduction is important for tracking and predicting changes in distribution, abundance, and population trends of ecologically and economically important consumers.

Our overall objective was to evaluate how macro-scale patterns in ungulate reproductive schedules vary with climatically driven plant phenology. To critically examine this relationship requires data systematically collected across large environmental gradients, which in our case extended over 8° of latitude (ca. 890 km). We analyzed extant data on mule deer reproductive parameters across our study region. Mule deer occupy a range of habitat types in western North America, and are subject to robust, systematic annual surveys that are comparable across jurisdictions. We evaluated three specific predictions with respect to mule deer reproduction and latitude. First, we predicted that mule deer birthing schedules would track local plant phenological signals, as measured using the Normalized Difference Vegetation Index (NDVI). This satellite-derived metric has proven useful in evaluating ungulate-forage relationships in a variety of systems [29, 30]. Second, we predicted that the mean and variance in juvenile production would be most sensitive to plant phenology (an index of forage quality) during the months of parturition [31]. Lastly, we predicted juvenile production would be highest at low latitudes defined by growing season precipitation, and conversely, lowest at higher latitudes where moisture regimes are dominated by winter snow and relatively dry summers. Here we highlight the costs and benefits to migratory mule deer associated with timing reproduction to plant phenology under different precipitation regimes.

## Materials and Methods

### Study species

The mule deer was selected as an appropriate taxon for evaluating these predictions for three reasons. First, it is a habitat generalist widely distributed throughout mountain ecosystems of western North America [32]; second, its reproductive parameters are sensitive to environmental variation [8]; and most importantly, as a common game species data are collected annually across most of its range using standardized methodology [33]. This enabled us to make comparisons over large spatial extents, and insure that results would be germane to species conservation at regional scales.

Mule deer reproductive physiology is defined by a 7-month gestation and a breeding strategy in which late gestation and lactation are supported through a combination of fat reserves and foraging behavior [8, 34]. Adult survival tends to be high and consistent, whereas juvenile survival displays considerable inter-annual variation [35, 36]. Mule deer typically perform seasonal migrations in areas where winter snowpack exceeds 45 cm [37]. Within the study region most sites above 2,200 m meet this threshold.

### Study region

**Fig 1:**
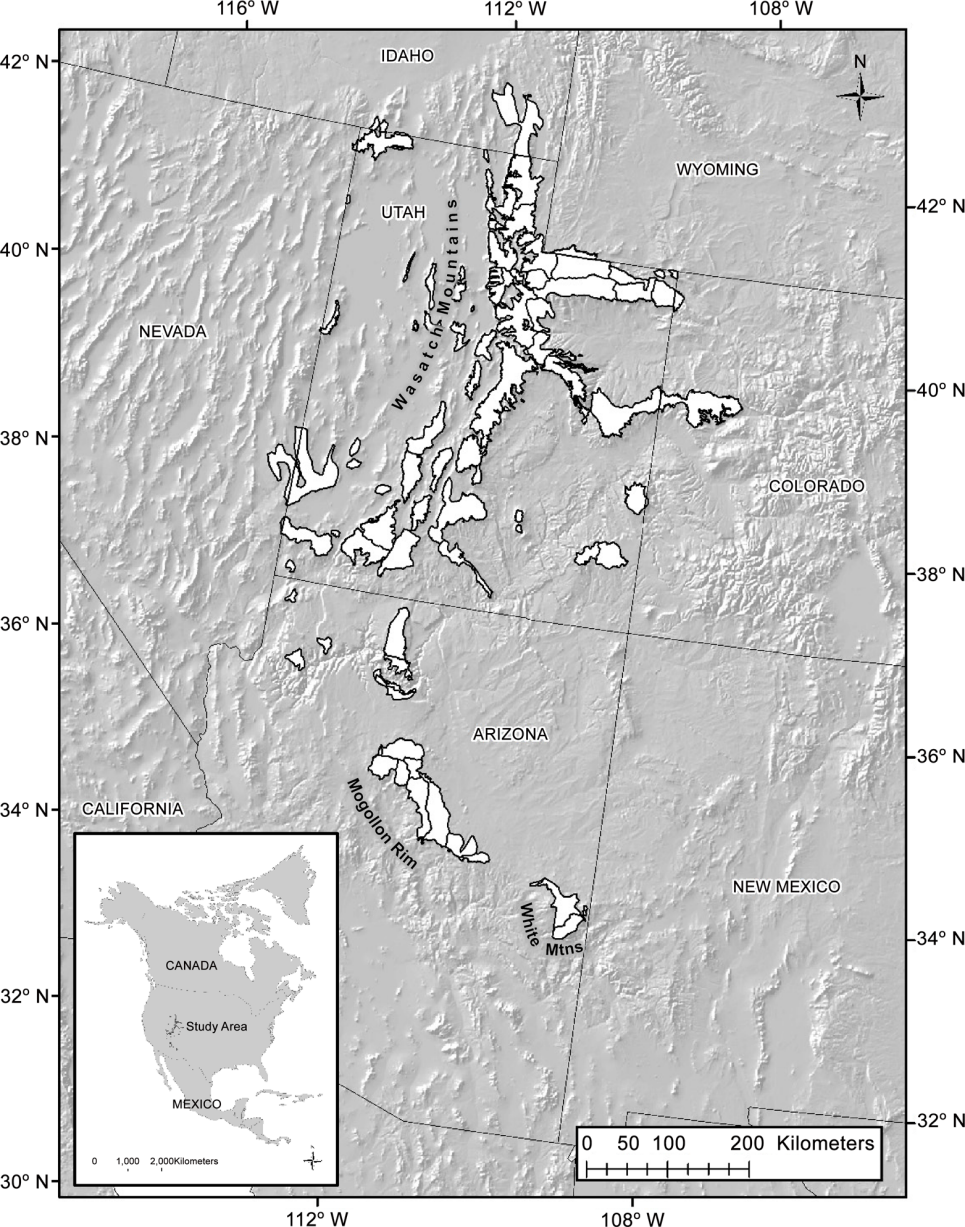
The study region spans portions of the Great Basin, Colorado Plateau, and Chihuahuan Desert ecoregions in southwestern North America (ca. 34–42° N). White polygons represent sampling units for all demographic and plant phenology data. Sampling units are based on the intersection of state-defined wildlife management units and high elevation summer mule deer habitat.

**Fig 2:**
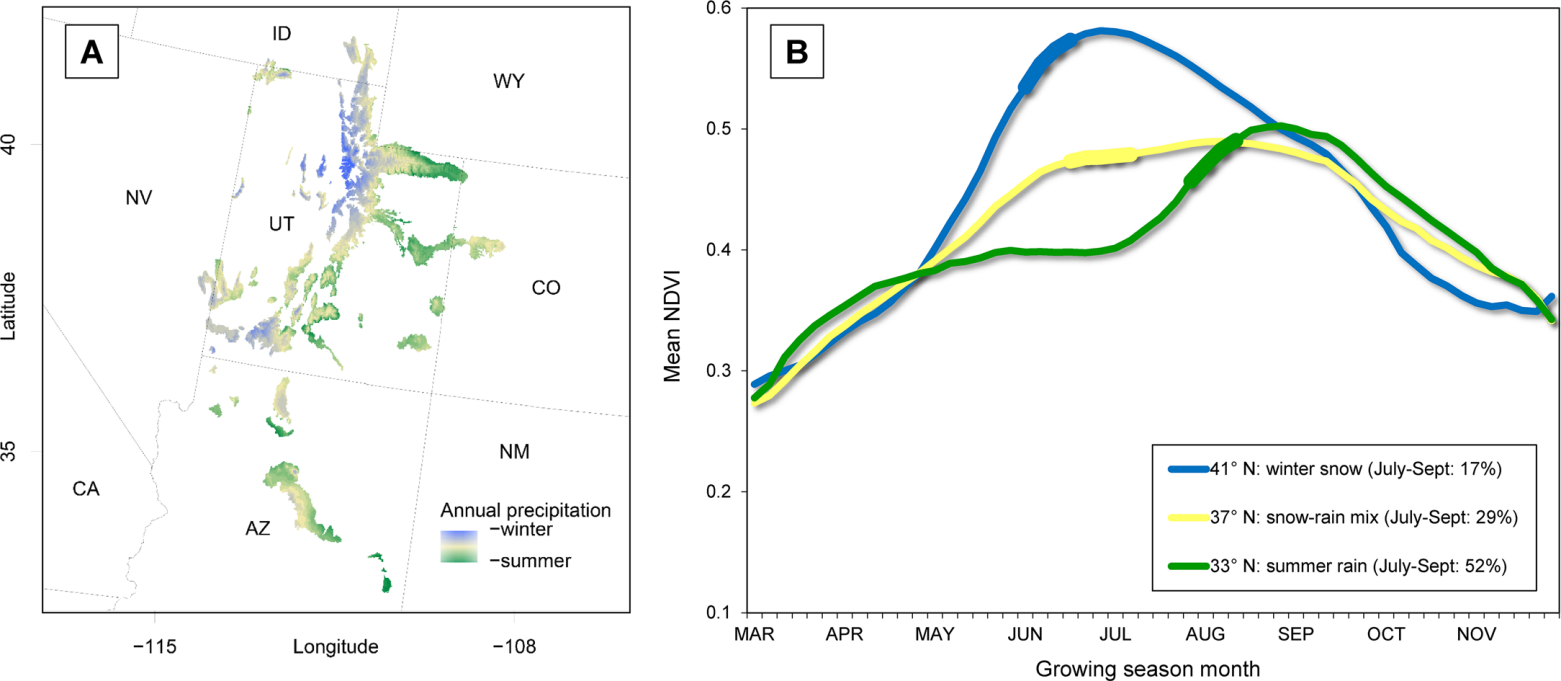
Panel A. Polygons symbolize high elevation summer mule deer habitats in southwestern North America (ca. 34–42° N). Map illustrates the latitudinal shift in the seasonality of moisture across the mountains of the Great Basin, Colorado Plateau, and northern Chihuahuan Desert ecoregions. Colors represent the percentage of total annual precipitation that comes in the form of thunderstorms during July-September (color key: blue ≤ 17%; beige = 18–49%; green ≥ 50%). Panel B. Growing season phenological curves for high elevation mule deer summer ranges in southwestern North America (2004–2011; 33–42° N). From top to bottom, curves represent a transition from ecosystems where plant phenology is driven by spring snowmelt and dry summers, to those in which phenology is timed to the onset of summer (monsoonal) rains. Thick bands represent approximate mule deer parturition dates at select latitudes.

We focused on high-elevation summer ranges extending from approximately 34° N, -114° W to 42° N -108° W (ca. 500 km x 890 km). This region extends from the White Mountains of central Arizona to the Wasatch Mountains of northern Utah and covers > 57,000 km^2^ (Fig 1). Climatically and botanically, the region represents a longitudinal transition zone between the Great Basin and Colorado Plateau ecoregions, with southern parts of the study region reflecting the influence of the Chihuahuan Desert. The region has a continental climate, defined by cold winters and hot summers, but more importantly, represents a latitudinal transition from mountains that derive the majority of their annual precipitation from winter-spring snowpack in the north, to those defined by growing season monsoons in the south [38]. In these monsoonal systems summer precipitation can account for > 40% of the total annual moisture budget (Fig 2). Across this gradient, botanical composition varies, but high elevation communities (> 2,200 m) where mule deer give birth are dominated by mixed conifer (*Picea sp*., *Abies sp*., *Pinus sp*.) and aspen (*Populus tremuloides*) forests. Intermediate elevations and drier aspects support piñon-juniper (*Pinus edulis*, *Juniperus sp*.) woodlands. At finer scales, sites that are disturbed, have shallow soils, or are located on warm aspects tend to support a mixture of grass and mountain shrub communities (*Artemisia tridentata*, *Purshia sp*., *Amelanchier sp*., and *Cercocarpus sp*.).

### Sampling units

Mule deer habitat, management unit boundaries, and demographic data were obtained from the Western States and Provinces Mule Deer Mapping Project, the Utah Division of Wildlife Resources (UDWR), and the Arizona Game and Fish Department (AZGF). To delineate summer birthing habitat we used the North American mule deer seasonal distribution map [39]. Summer habitat is defined here as that portion of the annual range where 90% of individual deer are located between spring green-up and the first heavy snowfall [39]. Habitat was mapped at the 1:250,000 scale based on expert opinion informed by extant survey, harvest, and radio-telemetry data. We extracted polygons representing summer habitat from the entire latitudinal gradient. Demographic data are collected on the basis of state-defined Wildlife Management Units (WMU), and so we intersected WMU polygons from Utah and Arizona with the summer habitat polygons to form the final sampling unit (Fig 1). Habitat polygons that contained agricultural fields, urban areas, and desert basins were excluded, as were portions of polygons extending into neighboring states or tribal lands. This was due to lack of data and/or confounding effects of anthropogenic subsidies on animal counts. Polygons ranged in size from 37–3,293 km^2^ (mean = 840 ± 709 km^2^) and from 1,635–3,121 m (mean = 2,332 ± 320 m) in elevation.

### Demographic variables

#### Parturition date

Date-of-birth (“Birthdate”) was estimated by meta-analysis of dates from migratory populations reported in peer-reviewed literature, reports from state wildlife agencies, and unpublished theses (S1 Appendix). Linear regression was used to predict Birthdate as a function of latitude from 34° to 42° N.

#### Fawn counts

We evaluated differences in mule deer productivity across the latitudinal gradient using the ratio of juveniles per 100 adult females (i.e. age ratios, or “fawn counts”). Age ratios are a simple and widely used metric for tracking productivity in ungulate populations [33]. Bonenfant et al. [40] cautioned that age ratios were subject to biases stemming from variation in sightability and animal behavior. Two independent evaluations however [41, 42], have validated the metric as a robust means of assessing relative differences in production under the proviso that sampling efforts are adequately replicated and temporally precise. Our data met these criteria. Two further points are worth noting. First, age ratios are the only data systematically collected over a broad enough geographic extent to allow evaluations at this scale; and second, these counts are one of the primary sources of data state agencies use to guide management decisions related to ungulate conservation. Here we used fawn counts and their associated variances as an index of broad scale spatial variation in net annual herd productivity.

Fawn counts are made at the end of the growing season and represent total annual reproductive output minus neonatal (summer) mortality. Adult mule deer exhibit high conception rates and relatively constant summer survival rates [36, 43], and so we assumed that differences in fawn counts across the gradient were primarily due to inherent differences in fetal rates and neonatal mortality. Importantly, latitudinal differences in winter severity may result in similar recruitment rates (i.e. first year survival), with the primary difference being when fawns succumb to various mortality factors during their first year. Thus, these data cannot be used to make inferences about overwinter survival or recruitment rates, but serve as an index of relative differences in annual production, or summer survival rates.

For all jurisdictions, fawn counts were conducted in December and January following the close of autumn hunting seasons. Survey data were derived using the methods outlined in [44, 45]. To ensure adequate replication we used only units with ≥ 6 years of data and > 300 animal observations / year / unit. Although harvest of antlerless animals can influence these ratios, opportunity for hunting females and yearlings is very limited in both states. In Arizona, only one of 14 units sampled offers an antlerless hunt (Kaibab 12A), whereas in Utah these hunts are localized around areas of agricultural conflict. Moreover, fawn surveys are designed to avoid anthropogenic landscapes specifically because they tend to be demographically biased. Sample sizes for annual mule deer surveys in Utah averaged 19,859 ± 3,147 (range = 16,862–25,097), and 2,862 ± 184 in Arizona (range = 2,493–3,305). The complete dataset spanned 2004–2011, and comprised > 180,000 animal observations [46, 47].

### Phenological variables

#### Normalized Difference Vegetation Index

We used NDVI [48, 49] both as an index of gross primary productivity, and as a means of measuring phenological events across the study region. This index has been used successfully to assess plant nutritional quality [29, 31] and model ungulate-forage relationships [30]. Estimates of red and near-infrared surface reflectance from the MODerate-resolution Imaging Spectroradiometer (MODIS) sensors aboard the Aqua and Terra satellites [50] were masked for snow, cloud, and high aerosols and then corrected for Bidirectional Reflectance Distribution Function (BRDF) effects using the Ross-Li-Magnan model [51]. BRDF parameters were estimated from an ensemble year (2000–2012) using a monthly moving window. Data gaps smaller than 16 days were filled using a locally weighted scatterplot smoothing (LOWESS) to produce a BRDF-corrected, daily, 500-m resolution series of red and near-infrared reflectance estimates in each pixel. This constituted the master dataset, or “stacks” from which other NDVI variables were derived.

From the master stacks we calculated five NDVI variables. The first was a multi-annual mean NDVI value for each polygon in our sampling frame for each month of the extended growing season (1 March to 30 November). The second variable was the mean date (day-of-year, or DOY) on which the highest NDVI value was recorded. This represented the peak of the growing season (or “Peak”). The inflection point in the spring growth curve has been identified as an important phenological event in ungulate ecology [52], and so our third NDVI variable was the mean date on which the inflection point occurred. This represented the typical date by which daily plant growth reached its apex. We interpreted this date as the start of the growing season (or “Start”), and measured it by calculating the date on which the first derivative of the spring growth curve reached its minimum (approximately zero). Here we defined spring as the period between the first snow-free day and the Peak. Importantly, in our study region the Start is more difficult to measure with precision than the Peak. This stems from two environmental factors; first, in northern regions detection of the emergence of green vegetation is hindered by the presence of cool-season grasses that germinate under snow. These semi-mature grasses are exposed during the spring melt, but are then repeatedly buried and exposed by late-season snowstorms. Second, summer ranges in the monsoon zone can have two growing seasons (spring, late summer), of which either can fail in any given year. Both of these phenomena confound the identification of the actual start of season. By comparison, the Peak is simply the highest NDVI value of the year and is relatively easy to measure with precision. Thus, some of the variability in Start dates may be an artifact of measurement error associated with that metric. Because of this, we have included both the Start and Peak in our analyses.

The standard deviations (SD) in the Start and Peak were the fourth and fifth variables. These variables represented the relative precision of the timing of these phenological events across latitude for the period 2000–2012.

Lastly, we calculated mean monthly precipitation for each sampling polygon in the study region using data from the PRISM Climate Group at Oregon State University (1981–2010) (http://prism.oregonstate.edu).

### Analytical techniques

To address our prediction that Birthdates would track phenological signals we evaluated relationships between the Birthdate, Start, and Peak using simple linear regression models and analysis of covariance (ANCOVA). We expected both the Start and Peak to occur later at higher latitudes, and that Birthdate would fall between the Start and Peak in time and track them across space. We included elevation as a covariate in the initial models because of its effect on seasonality. Stepwise procedures based on AIC were then used to determine if elevation should be retained.

We used ANCOVA to evaluate relationships between the Birthdate, Start, and Peak regression models. We first tested for an interaction effect using Birthdate, Start, and Peak as dummy factor levels; presence of an interaction would indicate that the slopes of the regression lines differed, and lead to the conclusion that these factors varied across latitude. Lack of interaction would indicate that Birthdate, Start, and Peak changed at a constant rate throughout the study region, and would require further tests to determine if the intercepts differed from one another. Lastly, we treated the SDs in the Start and Peak as response variables and used Spearman’s rank correlation coefficient (*r*) to determine if variation in the timing of those phenological events changed with latitude and summer precipitation.

Our second prediction was that fawn counts should be most sensitive to NDVI during the month of parturition. To evaluate this we created nine correlations between fawn count and mean NDVI for each growing season month (March–November). We calculated Spearman’s rank correlation coefficient for each of these models, and then plotted *r* as a response variable against each NDVI-month. We then repeated this procedure, using the coefficient of variation (CV) of fawn counts to calculate *r* values. The *r* values for both versions of the response variable were plotted together to illustrate the strength and direction (positive or negative) of the relationship between reproductive measures and NDVI over the course of the growing season. We then used analysis of variance (ANOVA) to statistically evaluate each growing season month as a predictor of the mean and CV in fawn counts.

Relative to the northern end of our study region, lower latitudes have milder winters and more growing-season precipitation. Thus, our third prediction was that mean juvenile production would be highest, and variance lowest, in southernmost habitats. We used linear regression to assess the relationship between both fawn count and its associated CV, and latitude.

All analyses using date as a response variable were performed on the day-of-year, but results are presented in day-month format for ease of interpretation. For all regressions we examined model residuals both formally (Shapiro-Wilk test) and visually through residual and qqnorm plots. These techniques provided a means of evaluating the veracity of underlying model assumptions, and where necessary, response variables were transformed to meet those assumptions. All analyses were conducted in R [53].

## Results

### Relationship between birthing schedules and plant phenological signals

**Fig 3:**
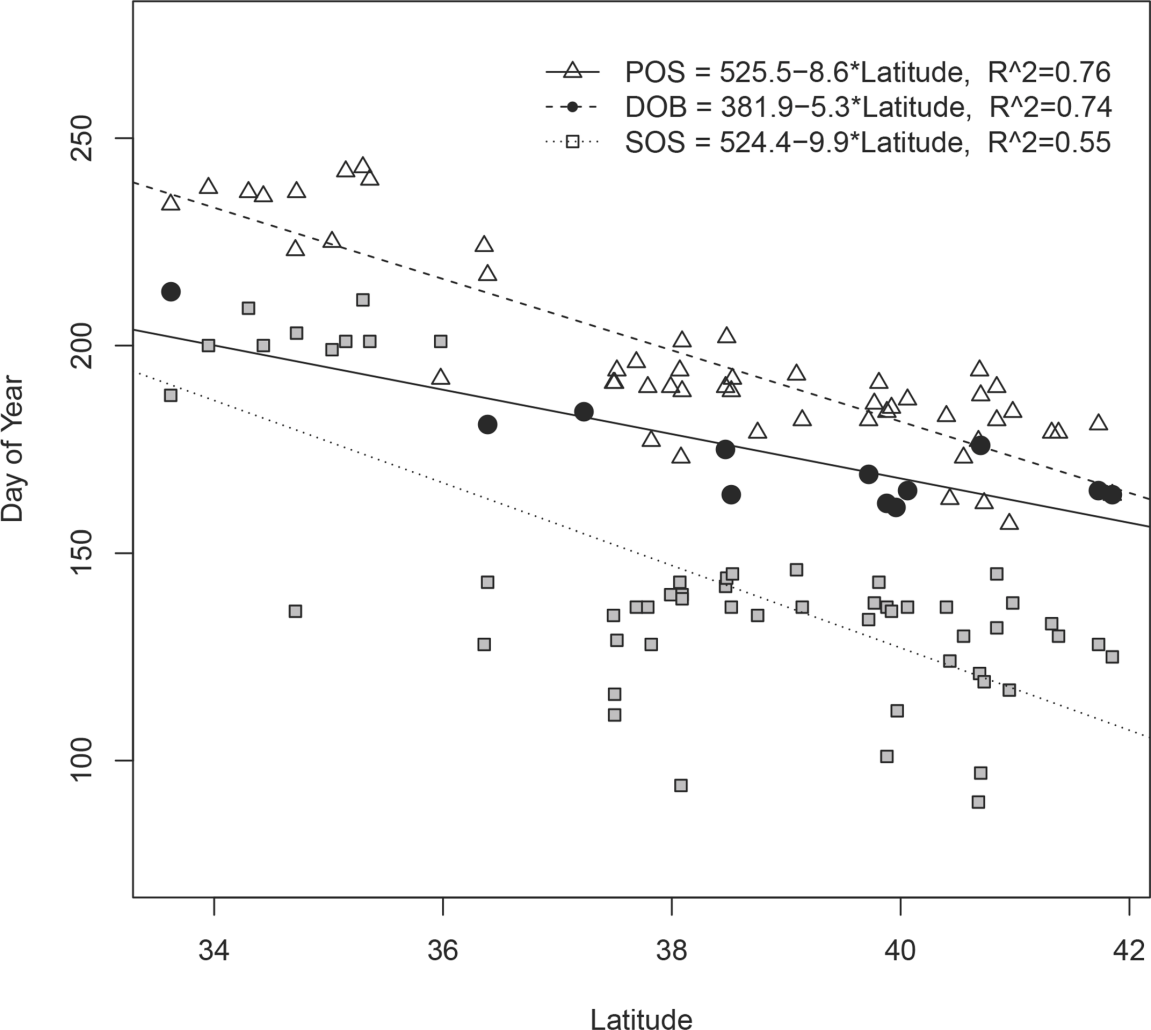
Estimated mule deer fawning dates (Birthdate), start of growing season (Start), and peak of growing season (Peak) as a function of latitude in southwestern North America, 2004–2011.

Models relating latitude to Birthdate, Start, and Peak, with and without elevation as an additional predictor, were all significant. However, stepwise comparisons indicated that the inclusion of elevation in the model provided no additional information (Birthdate model, *p* = 0.99; Start model, *p* = 0.79; Peak model, *p* = 0.42). We therefore retained only the simpler models using latitude as the single predictor variable. All models were all negatively related to latitude, with slopes of -5.35, -9.93, and -8.60, and r^2^ fits of 0.74, 0.55, and 0.76, for Birthdate, Start, and Peak, respectively (Fig 3).

**Fig 4:**
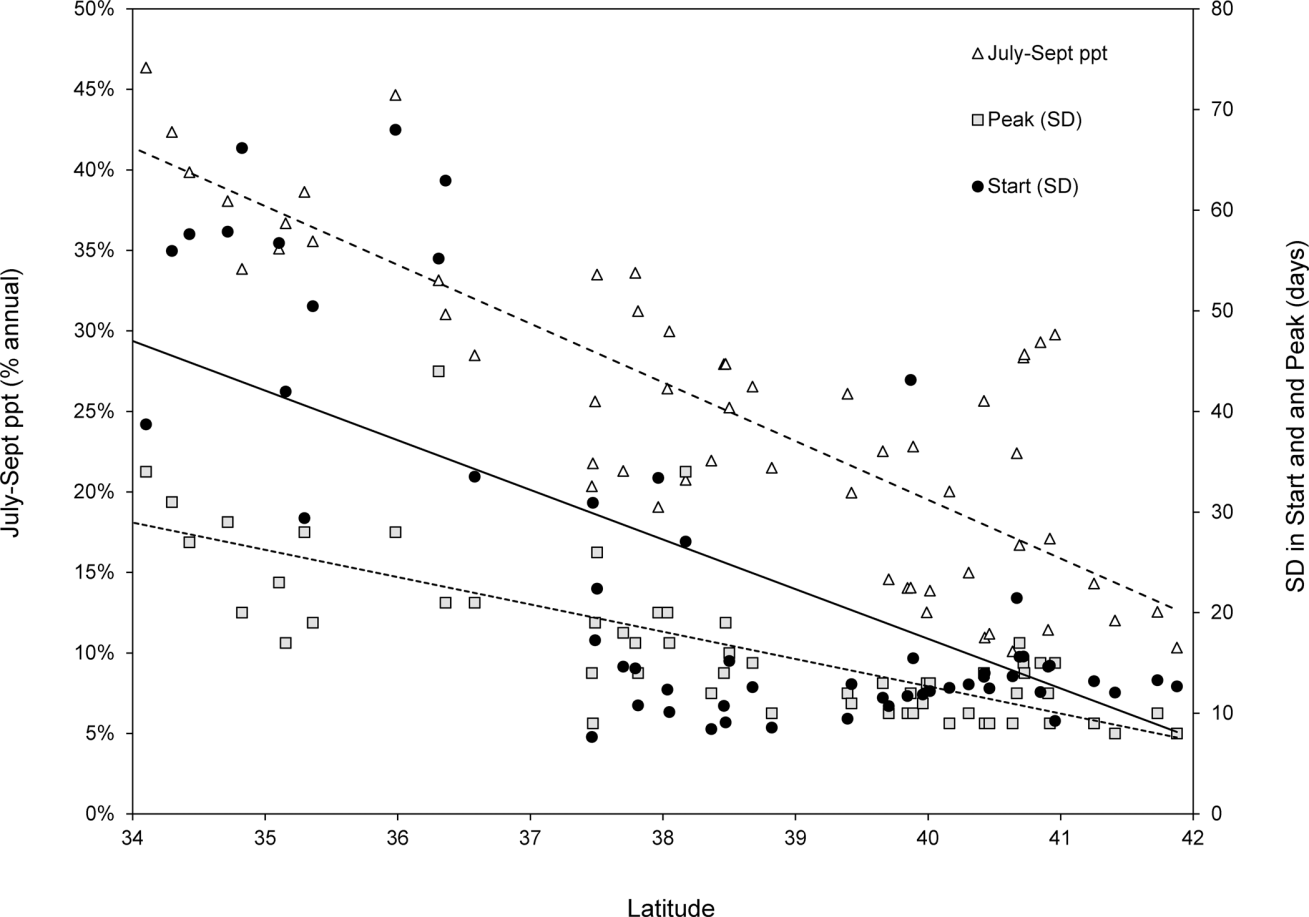
The proportion of annual precipitation occurring from July-September (monsoonal moisture) and the associated standard deviation (SD) in the start (Start) and peak (Peak) of season as a function of latitude in southwestern North America.

Contrary to expectation, peak plant productivity occurred earlier at higher latitudes (Fig 3). For units between 41–42° N, mean Start date was 114 (24 April ± 4 days), and 183 (4 July ± 3 days) for those between 34–35° N. The range for the region as a whole was 109 to 187 (19 April to 6 July). Mean Peak date was 170 at 41–42° N (19 June ± 3 days), and 230 at 34–35° N (18 August ± 3 days). The Peak ranged from 166 to 234 (15 June to 22 August). Precision of both Start and Peak varied with latitude. The SDs in Start and Peak ranged from 8–68, and 8–34 days, respectively, and both were negatively correlated with latitude (Spearman’s *r* = -0.54 and -0.76, respectively), but both metrics were positively correlated with monsoonal moisture, i.e. the proportion of total annual precipitation occurring from July-September (Spearman’s *r* = 0.44 and 0.80 for Start and Peak, respectively; Fig 4).

Birthdate occurred earlier with increasing latitude. Estimated dates ranged from 19 July at 34° N to 7 June at 41° N. Analysis of covariance indicated no interactions among the Birthdate, Start, and Peak models, and latitude (*df* = 2, *F* = 2.061, *p* = 0.132), suggesting that over this range of latitude, slopes of all models were statistically indistinguishable. However, all model intercepts were statistically different (*df* = 2, *F* = 137.4, *p* < 0.001), with the Start being lower (i.e., preceding) and Peak being greater (i.e., lagging) than Birthdate. On average, Birthdate occurred approximately 27 days after the Start and 23 days prior to the Peak.

### Relationship between juvenile production and plant phenology during parturition

**Fig 5:**
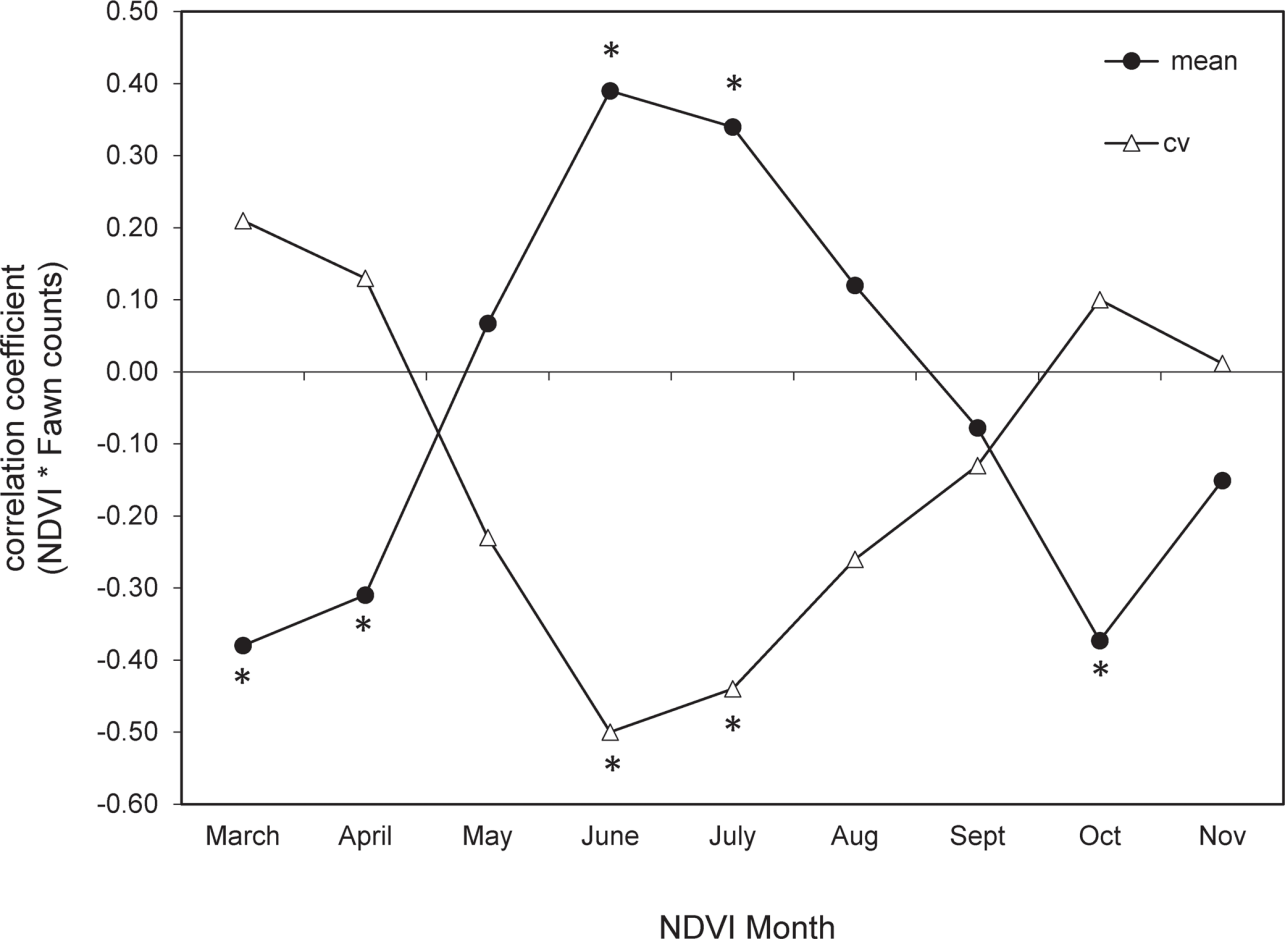
Values for Spearman’s rank correlation coefficient (*r*) between fawn counts and monthly NDVI values among migratory mule deer populations (2004–2011; 34–42° N). June and July are the primary months of parturition in this region. Asterisks indicate a statistically significant relationship between fawn counts and NDVI for that month.

Correlations between NDVI and the mean and CV in fawn counts, were strongest in June and July, which were the only two months that exhibited statistically significant relationships with both versions of the response variable (Table 1, Fig 5). Behaviorally, this period encompassed the interval spanning late pregnancy, parturition, and the early phases of lactation. The fawn- NDVI relationship was also significant during early spring (March-April) and late autumn (October). Notably, the CV (square root transformed) exhibited a significant, negative relationship with NDVI only during the extended birthing season.

**Table 1:** Relationship between the mean and CV (square root transformed) in mule deer fawn counts and mean NDVI, by growing season month, across a latitudinal gradient in southwestern North America (34°-42° N).

	mean (fawn counts)			CV (fawn counts)		
Month	r^2^	F	P	r^2^	F	P
**March**	0.17	11.6	**0.00**	0.05	2.8	0.10
**April**	0.12	7.8	**0.01**	0.02	1.3	0.25
**May**	0.01	0.4	0.50	0.06	3.7	0.06
June*	0.19	13.6	**0.00**	0.27	21.0	**0.00**
July*	0.13	8.7	**0.00**	0.20	14.6	**0.00**
**Aug**	0.01	0.3	0.59	0.05	3.2	0.08
**Sept**	0.01	0.8	0.39	0.01	0.5	0.48
**Oct**	0.15	10.2	**0.00**	0.02	1.0	0.33
**Nov**	0.03	2.1	0.16	0.00	0.0	0.85

Asterisks indicate months for which NDVI exhibited a statistically significant relationship with both versions of the response variable.

### Relationship between juvenile production and latitude

**Fig 6:**
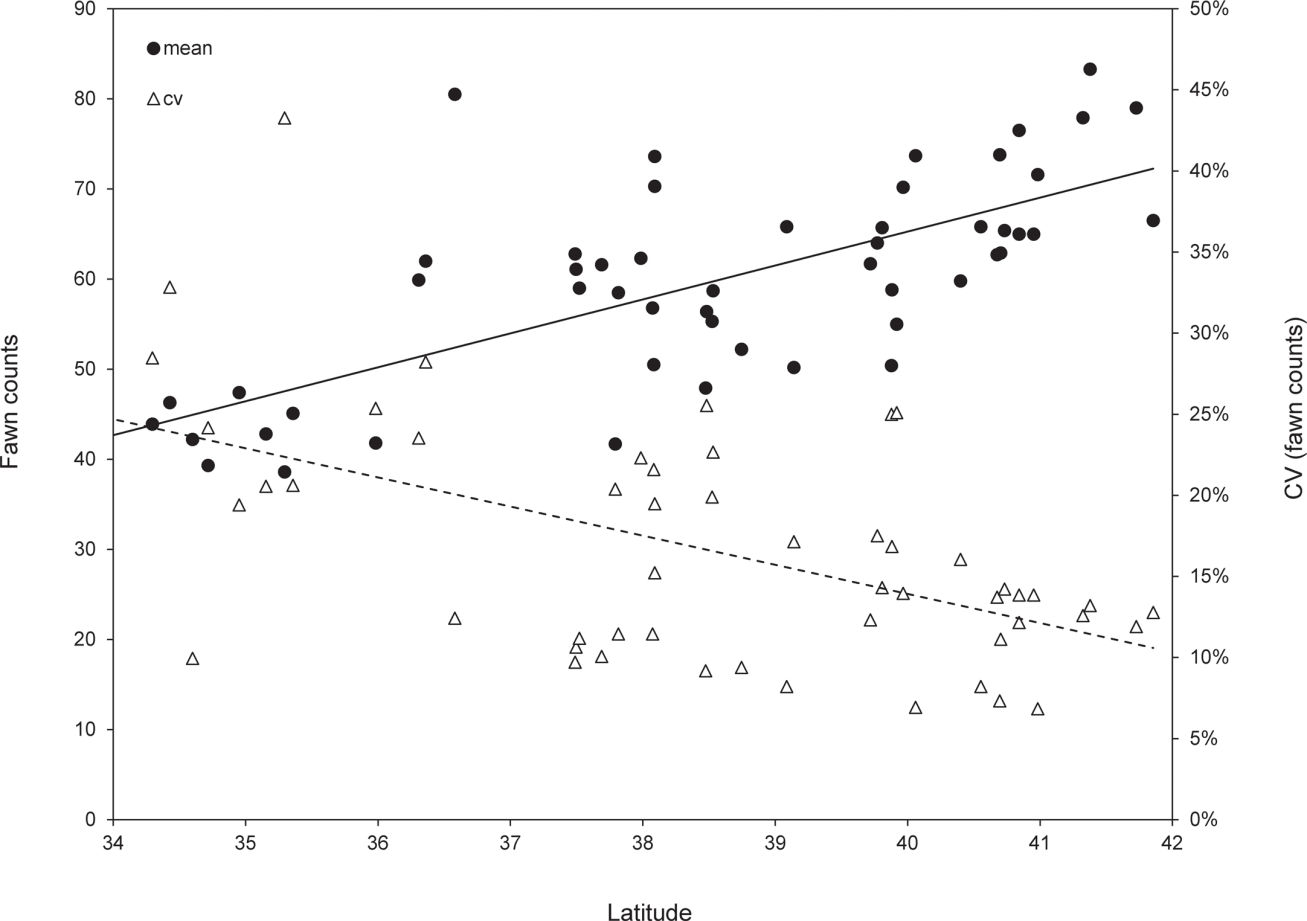
Mean autumn fawn counts and associated coefficient of variation (CV) for migratory mule deer populations in southwestern North America, 2004–2011.

Our prediction that juvenile production would be greater at lower latitudes was not borne out. Mean fawn counts decreased (y = 3.77x – 85.5; r^2^ = 0.56, *df* = 1, 52, *F* = 57.7, *p* < 0.001), and variation (ln transformed) increased (y = -0.1x + 1.9; r^2^ = 0.28; *df* = 1, 52, *F* = 20.2, *p* < 0.001) at lower latitudes (Fig 6).

## Discussion

### Do birthing schedules track local plant phenological signals?

Mule deer demonstrated a clear and significant reproductive response to phenological variation over a latitudinal and climatological gradient. In this region the Peak occurs in mid-June at higher latitudes, but gradually shifts to mid-August at lower latitudes. In accordance with our first prediction, mule deer gave birth from late May into July, with parturition dates following the Start and preceding the Peak across latitude. The lack of an interaction between Birthdate and either Start or Peak suggests that regardless of the underlying climatic drivers determining the timing of green-up (spring snowmelt vs. summer thunderstorms), mule deer tend to give birth approximately halfway between these phenological events. On average, this gap occurs approximately four weeks after the inflection point in the phenological growth curve, which corresponds to the highest nutritional value in green biomass [29, 31], suggesting that following migration, mule deer need 3–4 weeks of access to high quality forage in order to support the later stages of fetal growth. Timing parturition to the window between maximum plant nutrition (Start) and maximum forage availability (Peak) may be an important tactic for optimizing reproductive success when body condition and fat reserves are recovering from an annual low.

Our results corroborate earlier studies showing that parturient females compensate for rising energetic demands by increasing both the quality and amount of forage [5, 54, 55]. Importantly, our results demonstrate that this pattern is robust across precipitation regimes, and that as the climatological drivers of the Start and Peak change, ungulates incur a cost related to local variability in the timing of these phenological events. Indeed, the timing of the Start and Peak exhibits greater interannual variation under monsoonal conditions, than when triggered by spring snowmelt. As a consequence, mismatch may occur between the actual expression of the growing season and the mean Birthdate in any given year. Moreover, if higher temperatures associated with a late summer growing season lead to faster plant desiccation, then southern deer may have a shorter period of access to new growth during the lactation phase of reproduction, which can negatively influence juvenile survival [56].

Notably, mule deer differ from some high latitude (arctic) ungulates in this respect. Tveraa et al. [5] reported that Fennoscandian reindeer (*Rangifer tarandus*) give birth 2–4 weeks prior to the Start, but in that system the timing of Start was more predictable than the Peak. Among ungulates sympatric with mule deer, elk (*Cervus elaphus*) show little variation in parturition date with respect to local phenology [57], whereas bighorn sheep (*Ovis canadensis*) show a mixed strategy, in which only populations occupying subalpine or alpine habitats predictably time parturition to green-up [58]. In contrast, in desert environments bighorn sheep exhibit relatively low precision in birth timing, as mild winters and highly variable plant phenology reduces selection pressure on birth timing [58]. Thus, the strength of the relationship between parturition date and plant phenology is likely modified by the location of a given species along physiological gradients related to foraging and reproduction, i.e. concentrate selectors vs. grazers, and income vs. capital breeding strategies.

### Is juvenile production most sensitive to plant phenology during parturition?

Time-specific measures of NDVI have been used widely to evaluate interannual variation in ungulate demographic parameters [59, 60]. Results have underscored the influence of plant phenology during the perinatal period on body mass [5, 56, 59, 60], birth timing [5, 61, 62], and juvenile survival [56, 62],. Mean fawn counts demonstrated the strongest relationship with primary production around the time of parturition, but this association was also significant at the beginning and end of the growing season. In contrast to June-July, high NDVI in March-April was associated with low mean fawn counts. Many of the summer ranges sampled here are in the subalpine zone and can retain snowpack into June. Thus, high reflectance values in early spring may be an indicator of low snowpack and consequently low forage availability during the peak of season. The negative relationship with October NDVI is less clear, but is likely a sampling artifact stemming from the influence of mild weather on the timing and duration of the autumn migration. Demographic factors such as age and reproductive status have been associated with variation in the timing of autumn migration [63], and so fawn production may be underestimated in years when snowfall is delayed.

The relationship between the CV in fawn counts and NDVI was only significant during the period surrounding parturition, with marginal significance extending this effect to May-August. This suggests that units with high NDVI during reproductive months tend to exhibit lower interannual variation in fawn production. Spatially, these units were concentrated at higher latitudes, defined by greater winter precipitation and a green-up phase limited more by temperature than moisture. In general, results related to both mean and variation in fawn production support the hypothesis that current-year primary production has a strong influence on neonatal survival.

### Does juvenile production increase along a latitudinal gradient?

Relative to adult mule deer, juvenile survival tends to be lower and more variable [36]. In temperate mountain ecosystems, much of this variation is attributable to winter severity [64, 65]. Accordingly, we predicted that juvenile production would be highest at lower latitudes, as areas characterized by monsoonal moisture exhibit milder winters and greater amounts of growing season precipitation. Despite these climatic advantages, and contrary to our expectations, mule deer inhabiting the southernmost portions of our study region expressed lower annual production and higher interannual variation, than the snow-dominated systems to the north. That said, our analyses do not account for possible effects of density-dependence, which may partially explain differences in juvenile production, as the relative influence of density-dependent factors can vary with latitude [9].

Large herbivores respond indirectly to climate through vegetative phenology. In spring, green-up proceeds from equatorial regions poleward. However, local variation in this general pattern influences the ungulate reproductive schedules. The later Start and Peak at southern latitudes is the result of summer moisture playing an increasingly important role in plant phenology in those regions [66] (Figs 2 and 4). The relatively low fawn counts in southern habitats coincided with a progressively later and more variable Start and Peak. If deer miss the optimal window encompassing forage nutrition and / or availability, then variation in the timing and magnitude of these phenological events can translate into high interannual variability in fawn production, which effectively reduces the mean [22, 62]. The result of timing parturition to a monsoonal-driven growing season can be nutritional stress during the most demanding parts of the reproductive schedule. The relationship between environmental productivity, maternal nutrition, and juvenile survival has been demonstrated in other Great Basin and Rocky Mountain mule deer populations [8, 67], in which body condition predicted vulnerability to predation and annual survival rates for both juveniles and adults.

Along this latitudinal gradient, deer may be incurring biogeographic trade-offs with respect to prevailing climatic influences on the timing of reproduction. Mule deer inhabiting the northern third of our transect benefit from the relatively slow, predictable spring flush associated with snowpack-driven hydrology, but they assume a greater risk of winter malnutrition. Accumulating snow increases the energetic expenditures associated with travel, foraging, and thermoregulation [13]. Deer can mitigate some of these costs through migration, a common strategy for coping with food shortages in seasonal environments [17, 37, 63]. When compared to spring, autumn migration tends to be less synchronous, particularly during mild years, which allows longer access to relatively productive, high elevation plant communities. Indeed, autumn phenological curves have proven strong predictors of overwinter survival in mule deer [68]. In order to give birth, wean offspring, and accumulate fat reserves sufficient for autumn breeding and surviving the coming dormant season, migratory deer may time births to early summer in order to maximize the time their fawns have on summer range forage prior to fall migration. The cost of late birthing is that juveniles are more susceptible to winter stress, and maternal females may not recoup energetic losses associated with lactation prior to the onset of winter [15, 62]. Ultimately, recruitment is determined by overwinter survival, and therefore traits that influence this parameter are likely those under the highest selection pressure, of which the timing of conception and parturition are most important.

Winters become progressively milder at lower latitudes and so the timing and extent of migration may vary considerably from year to year [7]. Under these conditions selection pressure against late births is lighter and so overwinter survival is likely higher. However, relative to a spring flush, parturition calibrated to a monsoonal phenological signal means risking substantial interannual variation in the timing of Start and Peak. Monsoonal moisture is derived primarily from thunderstorms, which are spatially heterogeneous, and result in rapid run-off and high evaporation rates. In southern mountain habitats early snowmelt combined with a late-summer monsoon creates a drought condition during early summer, which coincides with the final stages of pregnancy. Nutritional stress during late gestation can result in underweight or malnourished neonates, and therefore lower summer survival [8].

Albon and Langvatn [17] noted that plant digestibility declined rapidly following germination and predicted that migratory ungulates would track the receding snowline in order to maximize access to high quality forage. In that sense, migration provides a means of reducing variability in diet quality through time. This general relationship has been validated through subsequent studies conducted on ungulate behavior in arctic, tropical, and temperate ecosystems [5, 18, 23, 52, 67, 69, 70]. Our study focused on migratory mule deer in a region characterized by high spatial and seasonal variability in precipitation. Across this climatological gradient deer timed parturition to the phenological window between the start and peak of the growing season. Although mean time between Start and Peak did not vary across latitude, variability in the onset of these measures did, with highest mean fawn production associated with those areas expressing a relatively consistent timing in the Start and Peak. Pettorelli et al. [56] hypothesized that longer exposure to emergent vegetation predicted higher juvenile survival. Given the high interannual variability associated with the monsoonal growing season, cohorts born during years with a weak monsoon, and consequently a brief growing season, would have limited access to high quality forage relative to their northern conspecifics. These patterns provide indirect support for this hypothesis.

Our findings offer a robust, parsimonious explanation for a focal parameter in ungulate demography across multiple degrees of latitude and contrasting climatological regimes. From an applied perspective, these models demonstrate the potential for broad-scale modeling of couplings between climate, plant phenology, and wildlife populations using space-borne observations. Monitoring phenological events has been proposed as a means of tracking climate change [4], and understanding animal responses to environmental variation is important for land-use planning, species conservation, and ecosystem management. Ungulate populations are monitored by state wildlife agencies because of the costs and benefits associated with recreational hunting, agricultural conflicts, and vehicle collisions [32, 71], and in arid systems, abundance tracks climatic fluctuations [24]. Climate projections for the American southwest suggest longer droughts and northward expansion of the monsoon [27, 72]. The link between ungulate life history and plant phenology across disparate systems [3, 5, 6], suggests that the integration of satellite imagery with *in situ* data collected systematically by natural resource agencies may provide a heretofore underutilized means of monitoring key demographic parameters of ungulate populations across a range of climatological modes. On longer time scales, the global coverage and fine temporal resolution of MODIS data offers a means of evaluating shifts in species distributions and abundance with respect to climate and land-use change (*sensu* [73, 74, 75]).

## Supporting Information

S1 Appendix Literature sources used to calculate date-of-birth (DOB) for migratory mule deer (*Odocoileus hemionus*) in the southwestern United States. (DOCX)
