# Supplementary material for: Ungulate Reproductive Parameters Track Satellite Observations of Plant Phenology across Latitude and Climatological Regimes
Source: PLoS One. 2016 Feb 5;11(2):e0148780. doi: 10.1371/journal.pone.0148780 (PMC4744036; doi:10.1371/journal.pone.0148780)
Supplement: S1 Appendix — (DOCX) [file pone.0148780.s001.docx]

**S1 Appendix.** Literature sources used to calculate date-of-birth (DOB) for mule deer (*Odocoileus hemionus*) in the southwestern United States.

No systematic assessment of parturition dates has been conducted for mule deer across their North American range. To evaluate variation in this parameter we conducted a review of the extant literature from the southwestern United States. In our assessment we included six measures from Utah [1, 2, 3, 8, 10, 11], two from Arizona [4, 9] and four from Colorado [3, 5, 6, 7]. The objectives of these studies varied, and so those focused on reproductive parameters produced the most precise estimates. Others, such as reviews [9] contained less methodological detail. Reported dates were presented either as a mean or a range, with ranges reported either quantitatively or qualitatively (e.g. 6/15 to 7/15 vs. “mid-June to mid-July”). To deal with this imprecision we developed a simple rule set for calculating dates. All citations used in our regression model are included in this Appendix. The bold number in parentheses at the end of each citation indicates the data category to which it belongs.

**Category (1)**. Direct quantitative estimates. These estimates were derived from studies using one or more of the following methods: (a) field observations of radio-marked females pre and post parturition, (b) age estimates of radio-marked neonates using morphometric-based regression models, or (c) age estimates of fetuses collected during necropsy of pregnant animals using morphometric-based regression models. All of these produced a mean value, which we used as the datum in our own regression model.

**Category (2)**. Quantitative estimates reported as a range. These were estimates based largely on field observations of unmarked animals. For either fawning or rutting dates presented as a range, we used the midpoint for our estimate. When only rutting (or conception) dates were presented, we added the mean gestation length of 203 days [8] to estimate the midpoint of the birth pulse.

**Category (3)**. Qualitative estimates reported as a range. Methods in these cases were unknown and likely derived from field surveys documenting rutting behavior and/or appearance of neonates. For either fawning or rutting dates presented as a range, we converted commonly used qualitative terms to numbers. For example, in citation [9] the author states, “Fawning occurs from June to August, reaching a peak in late June to early July.” We interpreted the qualitative terms “late” and “early” as one week, and “mid” as two weeks. Thus, the quotation above was converted to 23 June to 7 July, producing a point estimate of 30 June, the value used in the regression model.

Lastly, we used elevation and latitude as predictor variables in the regression equation. We obtained these values from study area descriptions in the original sources. When these were lacking, we estimated values from the centroid of each study area using digital elevation models in ArcGIS.

1. Berryman JH. Mammals of the Raft River Mtns, Utah [Thesis]. Salt Lake City (UT): University of Utah; 1948. **(3)**
2. Evans T. A study of the Rocky Mtn Mule deer *(O. h. macrotis*) Say, in northern Utah [Thesis]. Logan (UT): Utah State Agricultural College; 1939. **(2)**
3. Freeman ED, Larsen RT, Peterson ME, Anderson Jr. CR, Hersey KR, McMillan BR. Effects of male-biased harvest on mule deer: implications for rates of pregnancy, synchrony, and timing of parturition. Wildlife Society Bulletin. 2014; 38:806-811. **(1)**
4. Heffelfinger J. Deer of the Southwest. College Station (TX): Texas A & M University Press; 2006. **(NA)**
5. Medin DE, Anderson AE. Modeling the dynamics of a Colorado mule deer population. Wildlife Monographs. 1979; 68: 3-77. **(2)**
6. Mierau, GW, Schmidt JL. Mule deer of Mesa Verde National Park. Mesa Verde Research Series, paper no. 2; 1981 (data presented in Table 13 of Heffelfinger, 2006). **(2)**
7. Robinette, WL, Baer CH, Pillmore RE, Knittle CE. Effects of nutritional change on captive mule deer. Journal of Wildlife Management. 1973; 37: 312-326. **(1)**
8. Robinette WL, Hancock NV, Jones DA. The Oak Creek mule deer herd in Utah. Salt Lake City (UT): Utah Division of Wildlife Resources; 1977. Publication no. 77-15. **(1)**
9. Russo JP. The Kaibab north deer herd: it's history, problems, and management. Phoenix (AZ): Arizona Game and Fish Department; 1964. Wildlife Bulletin no. 7. **(3)**
10. Smith RB. Mule deer reproduction and survival in the La Sal Mtns, Utah [Thesis]. Logan (UT): Utah State University; 1983. **(1)**
11. Zwank P. Reduced recruitment in Utah mule deer relative to winter condition. Salt Lake City (UT): Utah Division of Wildlife Resources; 1979. Publication 79-11. **(1)**
